# Renal dysfunction improves risk stratification and may call for a change in the management of intermediate- and high-risk acute pulmonary embolism: results from a multicenter cohort study with external validation

**DOI:** 10.1186/s13054-021-03458-z

**Published:** 2021-02-09

**Authors:** Romain Chopard, David Jimenez, Guillaume Serzian, Fiona Ecarnot, Nicolas Falvo, Elsa Kalbacher, Benjamin Bonnet, Gilles Capellier, François Schiele, Laurent Bertoletti, Manuel Monreal, Nicolas Meneveau

**Affiliations:** 1 Department of Cardiology, University Hospital Jean Minjoz, 3 Boulevard Fleming, 25000 Besançon, France; 2 EA3920, University of Burgundy Franche-Comté, Besançon, France; 3 Respiratory Department, Hospital Ramón y Cajal and Instituto Ramón y Cajal de Investigación Sanitaria (IRYCIS), Madrid, Spain; 4 Department of Medicine, Universidad de Alcalá (IRYCIS), Madrid, Spain; 5 Department of Internal Medicine, University Hospital Dijon-Bourgogne, Dijon, France; 6 Medical Oncology Unit, University Hospital Besançon, Besançon, France; 7 Department of Cardiology, General Hospital, Vesoul, France; 8 Medical Intensive Care Unit, University Hospital Jean Minjoz, Besançon, France; 9 Department of Vascular and Therapeutic Medicine, Saint-Etienne University Hospital, Saint-Etienne, France; 10 INSERM CIC1408 and INSERM UMR 1059, Saint-Etienne, France; 11 F-CRIN, INNOVTE, Saint-Etienne, France; 12 Department of Internal Medicine, Hospital Universitari Germans Trias I Pujol, Badalona, Barcelona Spain

**Keywords:** Pulmonary embolism, Renal dysfunction, All-cause death, Stratification

## Abstract

**Background:**

Renal dysfunction influences outcomes after pulmonary embolism (PE). We aimed to determine the incremental value of adding renal dysfunction, defined by estimated glomerular filtration rate (eGFR), on top of the European Society of Cardiology (ESC) prognostic model, for the prediction of 30-day mortality in acute PE patients, which in turn could lead to the optimization of acute PE management.

**Methods:**

We performed a multicenter, non-interventional retrospective post hoc analysis based on a prospectively collected cohort including consecutive confirmed acute PE stratified per ESC guidelines. We first identified which of three eGFR formulae most accurately predicted death. Changes in global model fit, discrimination, calibration and reclassification parameters were evaluated with the addition of eGFR to the prognostic model.

**Results:**

Among 1943 patients (mean age 67.3 (17.1), 50.4% women), 107 (5.5%) had died at 30 days. The 4-variable Modification of Diet in Renal Disease (eGFR_MDRD4_) formula predicted death most accurately. In total, 477 patients (24.5%) had eGFR_MDRD4_ < 60 ml/min. Observed mortality was higher for intermediate–low-risk and high-risk PE in patients with versus without renal dysfunction. The addition of eGFR_MDRD4_ information improved model fit, discriminatory capacity, and calibration of the ESC model. Reclassification parameters were significantly increased, yielding 18% reclassification of predicted mortality (*p* < 0.001). Predicted mortality reclassifications across risk categories were as follows: 63.1% from intermediate–low risk to eGFR-defined intermediate–high risk, 15.8% from intermediate–high risk to eGFR-defined intermediate–low risk, and 21.0% from intermediate–high risk to eGFR-defined high risk. External validation in a cohort of 14,234 eligible patients from the RIETE registry confirmed our findings with a significant improvement of Harrell’s C index and reclassification parameters.

**Conclusion:**

The addition of eGFR_MDRD4_-derived renal dysfunction on top of the prognostic algorithm led to risk reclassification within the intermediate- and high-risk PE categories. The impact of risk stratification integrating renal dysfunction on therapeutic management for acute PE requires further studies.

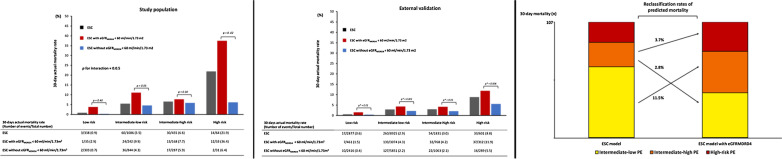

## Background

Risk stratification of early mortality in acute pulmonary embolism (PE) represents an important step in assessing individual prognosis and in therapeutic decision making [1]. Low- and intermediate–low-risk PE should be treated promptly with anticoagulant therapy. Intermediate–high-risk PE needs additional close monitoring in the intensive care unit (ICU) to detect secondary hemodynamic collapse. High-risk PE requires urgent pulmonary revascularization therapy to restore pulmonary flow and improve right ventricular (RV) function [1].

Patients with renal dysfunction have an increased incidence of venous thromboembolism (VTE) compared to patients with normal renal function [2]. The prevalence of reduced renal function was as high as 40% in a medical records review including more than 6700 VTE patients [3]. Furthermore, evidence is mounting that renal dysfunction is associated with death after PE [4–6]. The European Society of Cardiology (ESC) guidelines for the management of PE introduced severe renal dysfunction (i.e., estimated glomerular filtration rate (eGFR) < 30 ml/min), which is included in the Hestia checklist, as a possible criterion to guide the choice of home treatment or hospitalization in low-risk patients [1, 7].

However, we hypothesize that renal dysfunction may be predictor of death that is sufficiently powerful to improve the accuracy of risk algorithms, which in turn could lead to the optimization of acute PE management.

Therefore, we sought to investigate the incremental value of adding renal dysfunction on top of the ESC-defined prognostic algorithm for the prediction of 30-day mortality in patients with acute PE from a multicenter cohort study. To this end, we first identified which of the three formulae available for eGFR calculation, namely the four-variable Modification of Diet in Renal Disease (MDRD4), the Chronic Kidney Disease Epidemiology Collaboration (CKD-EPI), or the Cockcroft–Gault (CG) equations [8], predicted death most accurately. We performed an external validation of the incremental prognostic value of renal dysfunction using data from the Registro Informatizado de la Enfermedad TromboEmbólica (RIETE registry).

## Methods

This cohort study is a non-interventional retrospective post hoc analysis based on prospectively collected data from five French centers (two tertiary care facilities and three general hospitals) between September 2012 and November 2019, and recorded in the Burgundy Franche-Comte (BFC)-FRANCE registry [9]. This registry received approval from the national commission for data privacy and protection. This study was conducted in accordance with the amended Declaration of Helsinki. All patients provided written informed consent, and our institutional review board approved the study. We report the study methods and results in accordance with the STrengthening the Reporting of OBservational studies in Epidemiology (STROBE) guidelines [10].

### Patients and setting

We prospectively recorded all consecutive patients ≥ 18 years with: (1) a confirmed diagnosis of PE by computed tomography pulmonary angiography [11], or ventilation-perfusion scan [12], and (2) RV function assessment by echography at admission according to the guidelines [1]. There were no exclusion criteria. Management was at the discretion of the physician in charge. All sites followed a standardized protocol for data collection, as previously described [9].

### Estimated glomerular filtration rate

Blood samples were collected at admission. GFR was estimated with MDRD4, CKD-EPI, and body surface-adjusted CG (CG-BSA) equations (Additional file 1: Table S1) [8]. We defined renal dysfunction as eGFR < 60 ml/min according to the KDIGO guidelines [13].

### Early risk stratification

Pulmonary embolism was risk-stratified according to the ESC guidelines as low, intermediate–low, intermediate–high, and high risk [1]. Patients with cardiac arrest, obstructive shock, or persistent hypotension were stratified as high risk. Hemodynamically stable patients with simplified Pulmonary Embolism Severity Index (sPESI) ≥ 1, RV dysfunction, and positive troponin were stratified as intermediate–high risk. Hemodynamically stable patients with sPESI ≥ 1 and/or RV dysfunction were stratified as intermediate–low risk. The remaining hemodynamically stable patients with sPESI = 0, normal RV function, and negative troponin were stratified as low risk.

### Outcomes and definitions

The primary outcome was death from any cause within 30 days after admission for acute PE. The secondary outcome was major bleeding or clinically relevant non-major bleeding defined according to the definition of the International Society of Thrombosis and Haemostasis [14]. All suspected outcome events were classified by a central adjudication committee (GS and FS). Disagreement was resolved by a third author (RC). PE was considered as the cause of death if there was objective documentation or if death could not be attributed to another documented cause and PE could not be ruled out. Hemodynamic instability includes obstructive shock, defined as systolic blood pressure (BP) < 90 mmHg or vasopressors required to achieve a BP ≥ 90 mmHg despite an adequate filling status, in combination with end-organ hypoperfusion; or persistent hypotension, defined as systolic BP < 90 mmHg or a systolic BP drop ≥ 40 mmHg for > 15 min [1]. Positive troponin was defined as a value > 99th percentile of healthy subjects with a coefficient of variation of 10%. RV dysfunction was defined as the presence of at least one of the following on admission echography: peak systolic gradient at the tricuspid valve > 30 mmHg, increased end-diastolic RV/left ventricle diameter ≥ 1.0 in the apical four-chamber view, flattened intraventricular septum, decreased tricuspid annular plane systolic excursion < 16 mm, or right heart thrombus detected in right heart cavities [1].

### Statistical analysis

Continuous variables are expressed as mean (standard deviation). Categorical variables are expressed as number (percentage). Unadjusted differences between kidney function groups were compared using the Chi-square test or Student’s t test as appropriate. Multivariable analyses were performed by logistic regression, adjusted for baseline characteristics, and in-hospital therapies that yielded a *p* value < 0.10 by univariable analysis. Results are reported as odds ratio (OR) with 95% confidence interval (CI).

The eGFR equation that best-predicted mortality was selected by calculating Harrell’s C-indices, integrated discrimination improvement (IDI), and net reclassification improvement (NRI) for each survival model including eGFR as a categorical variable [15–17]. Crude incidences of bleeding across each PE risk category between patients with and without renal dysfunction were estimated using the cumulative incidence function and compared using the Gray test.

To assess the incremental value of adding renal function information on top of the ESC prognostic algorithm, we used the following approaches [18, 19]: (1) changes in measure of overall fit; (2) changes in indices of calibration (i.e., Hosmer–Lemeshow parameters); (3) changes in indices of discrimination (i.e., Harrell’s C-statistic); and (4) changes in predicted risk reclassification by calculating the IDI, continuous NRI, and user category NRI [20].

To ensure that our findings were reproducible, we performed an external validation based on data from the RIETE registry [21]. The validation cohort for this study consisted of a group of 14,234 patients with acute symptomatic PE, and available creatinine and echographic data at admission.

A *p* value < 0.05 was considered significant. Analyses were performed using SAS 9.4 (SAS institute Inc., Cary, NC).

## Results

### Study sample

**Fig. 1:**
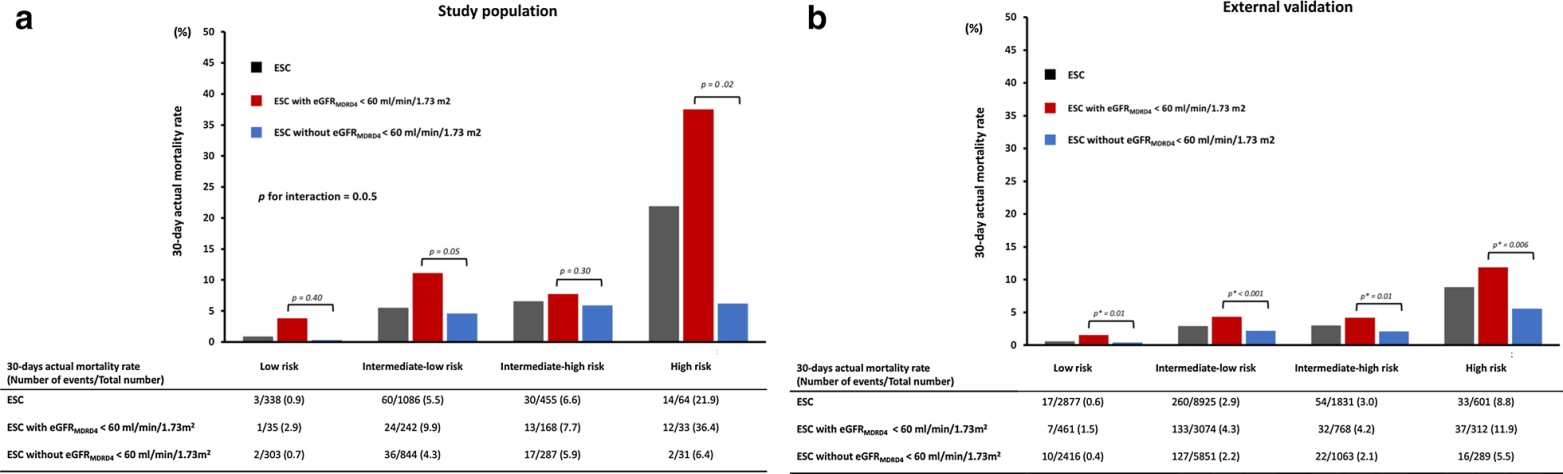
Bar chart of observed 30-day all-cause mortality by risk categories from a prognostic algorithm with and without renal function impairment (i.e., eGFR_MDRD4_ < 60 ml/min), in the study population (**a**) and in the RIETE population for external validation (**b**). eGFR_MDRD4_: estimated glomerular filtration rate calculated with four-variable Modification of Diet in Renal Disease equation. Interaction between renal dysfunction and risk categories was tested by the Breslow–Day test. *unadjusted *p* value. *p* value for interaction: overall comparison of the difference in observed mortality between patients with and patients without eGFR_MDRD4_ < 60 ml/min across risk groups

Of the 2102 patients admitted with an objective diagnosis of acute PE during the study period, 8 (0.4%) were lost to follow-up. Among the remaining 2094 patients, eGFR and echographic data were unavailable for 151 (7.6%). The eligible study cohort thus included 1943 patients (92.4%). Mean age was 67.3 (17.1) years, 50.4% were women (Table 1). Sixty-four patients (3.3%) had high-risk PE, 455 patients (23.4%) intermediate–high-risk PE, 1086 patients (55.9%) intermediate–low-risk PE, and 338 patients (17.4%) low-risk PE (Fig. 1).

**Table 1:** Baseline characteristics and management of the study population (*n* = 1943) according to the estimated glomerular filtration rate calculated with the four-variable Modification of Diet in Renal Disease (eGFR_MDRD4_) formula

Variables	Total (*n* = 1943)	eGFR_MDRD4_ < 60 ml/min		*p* value
		Yes (*n* = 477)	No (*n* = 1466)	
Age, years	67.3 ± 17.1	77.3 ± 11.9	64.1 ± 17.3	< 0.001
Male (%)	962 (45.5)	182 (38.2)	780 (53.2)	< 0.001
BMI (kg/m^2^)	27.4 ± 5.5	28.0 ± 6.2	27.1 ± 5.8	0.007
Site of care				
Conventional ward	1328 (68.3)	251 (52.6)	1077 (73.4)	–
Intensive care unit	615 (31.6%)	226 (47.3)	389 (26.5)	–
Comorbidities (%)				
Coronary disease	282 (14.5)	109 (22.8)	173 (11.8)	< 0.001
Pulmonary disease/HF	174 (8.5)	54 (11.3)	120 (8.2)	0.03
Active cancer	367 (18.9)	84 (17.6)	283 (19.3)	0.41
Prior VTE	474 (24.3)	115 (24.1)	359 (24.5)	0.86
Prior bleeding	67 (3.4)	26 (3.5)	41 (3.4)	0.87
Low risk for long-term VTE recurrence (%)	486 (25.0)	113 (23.7)	373 (25.4)	0.44
Associated DVT (%)	794 (40.8)	195 (40.9)	599 (40.8)	0.99
Clinical characteristics				
HR at admission (bpm)	89.6 ± 19.3	89.7 ± 19.5	89.6 ± 19.2	0.92
SBP at admission (mmHg)	137.1 ± 22.3	135.5 ± 26.2	138 ± 21.9	0.03
SaO^2^ (%)	93.4 ± 23.7	92.9 ± 5.4	93.9 ± 4.9	< 0.001
Biological data				
Hemoglobin (g/dL)	13.2 ± 2.6	12.8 ± 2.27	13.5 ± 3.4	< 0.001
Positive troponin	853 (43.9)	308 (564.6)	545 (37.2)	< 0.001
Echo data				
sPAP (mmHg)	42.7 ± 15.8	47.9 ± 15.7	40.6 ± 15.3	< 0.001
RV dysfunction^a^	800 (41.2)	258 (54.1)	542 (36.9)	< 0.001
Peak systolic gradient at the tricuspid valve > 30 mmHg	639 (32.9)	218 (45.7)	421 (28.7)	< 0.001
End-diastolic RV/LV diameter ≥ 1.0 in the apical four-chamber view	291 (15.0)	112 (23.5)	179 (12.2)	< 0.001
Flattened intraventricular septum	267 (13.7)	99 (20.7)	168 (11.5)	< 0.001
TAPSE < 16 mm	85 (4.4)	33 (6.9)	52 (3.5)	0.002
Right heart thrombus	26 (1.3)	11 (2.3)	15 (1.0)	0.03
sPESI (points, Q1-Q3)	1 (1–2)	2 (1–3)	1 (0–2)	< 0.001
In-hospital treatments (%)				
Reperfusion therapy				
Thrombolysis	93 (4.8)	43 (9.0)	50 (3.4)	< 0.001
Surgical embolectomy	9 (0.5)	5 (1.0)	4 (0.3)	0.03
ECMO	9 (0.5)	7 (1.5)	2 (0.1)	< 0.001
Inferior vena cava filter	8 (0.4)	3 (0.6)	5 (0.3)	0.39
Outpatient treatment^b^	28 (1.4)	7 (1.4)	21 (1.4)	1.0
Early discharge^c^	198 (10.2)	38 (8.0)	160 (10.9	0.07

BMI, body mass index; VTE, venous thromboembolic; DVT, deep vein thrombosis; PE, pulmonary embolism; HR, heart rate; SBP, systolic blood pressure; SaO2, arterial oxyhemoglobin saturation; RV, right ventricle; LV, left ventricle; sPAP, systolic pulmonary arterial pressure; LV, left ventricle; TAPSE, tricuspid annular plane systolic excursion; ECMO, extracorporeal membrane oxygenation

^a^Defined by the presence of at least one of the echographic parameters below

^b^Pulmonary embolism management from the emergency room to home

^c^Pulmonary embolism management with discharge at day one

In total, 477 patients (24.5%) had an eGFR_MDRD4_ < 60 ml/min, 547 (28.1%) had an eGFR_CKD-EPI_ < 60 ml/min, and 736 (37.9%) had an eGFR_CG-BSA_ < 60 ml/min at admission. Overall, patients with renal dysfunction were older, had more comorbidities, and more severe hemodynamic profile at admission, whatever the formula used for eGFR calculation (Table 1 and Additional file 1: Table S2).

**Fig. 2:**
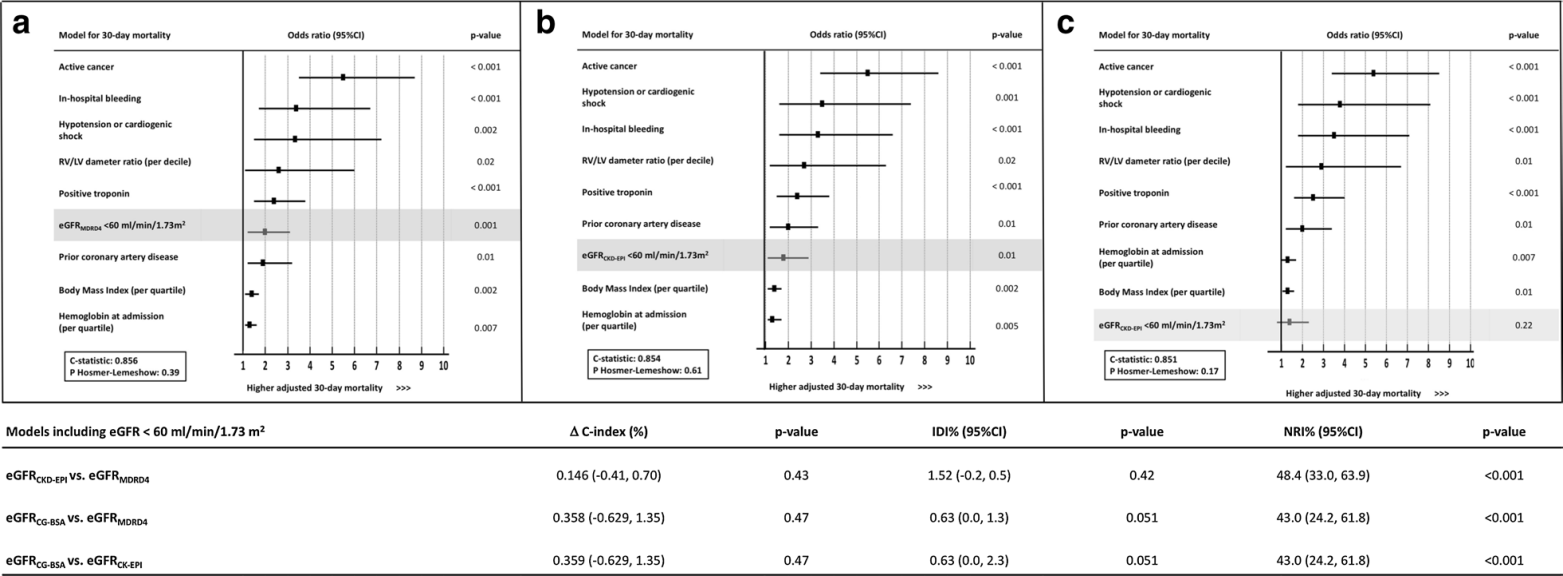
Forrest plot of adjusted variables associated with 30-day all-cause mortality after acute pulmonary embolism in the study population (*n* = 1943) (Model A with eGFR_MDRD4_, model B with eGFR_CKD-EPI_, model C with eGFR_CG-BSA_), and the associated table displaying comparisons between estimated glomerular function rate (eGFR) equations. MDRD4: four-variable Modification of Diet in Renal Disease equation; CKD-EPI: Chronic Kidney Disease Epidemiology Collaboration equation; CG-BSA: body surface area-adjusted Cockcroft–Gault equation; RV: right ventricle, LV: left ventricle; CAD: coronary artery disease; BMI: body mass index; IDI: integrated discrimination improvement; NRI: net reclassification improvement; CI: confidence intervals

One hundred seven out of 1943 patients (5.5% (95% CI 4.5–6.5%)) died during the 30-day follow-up. Mean time from admission to all-cause death was 3.2 (1.3) days. Causes of death were: PE (47.0%), cancer (23.2%), bleeding (12.1%), and other causes (17.7%). The 30-day mortality rates for patients with versus without eGFR-derived renal dysfunction were: 10.4% versus 3.9% for the eGFR_MDRD4_ groups, 9.9% versus 3.8% for the eGFR_CKD-EPI_ groups, and 8.8% versus 3.5% for the eGFR_CG-BSA_ groups. Variables significantly associated with 30-day mortality by univariable analysis are shown in Additional file 1: Table S3. After multivariable adjustment, eGFR_MDRD4_ and eGFR_CKD-EPI_-defined renal dysfunction were significantly associated with 30-day mortality, whereas eGFR_CG-BSA_ was not (Fig. 2).

Additional file 1: Table S3 displays covariates significantly associated with 30-day major bleeding by univariable analysis (see Additional file 1). The adjusted major bleeding rates at 30 days were higher in patients with renal dysfunction whatever the formula used for eGFR calculation (6.9% vs 3.0%, *p* = 0.004, with eGFR_MDRD4_; 7.1% vs 2.7%, *p* = 0.007, with eGFR_CKD-EPI_; 5.8% vs 2.8%, *p* = 0.04, with eGFR_CG-BSA_, and in patients with age > 75 years, prior stroke, hypotension cardiogenic shock, and positive troponin at admission.

### eGFR formulae for mortality prediction.

Discriminatory (Harrell’s C index) and reclassification (IDI and NRI) parameters were higher with the 30-day mortality model including the eGFR_MDRD4_-defined renal dysfunction than in those including renal dysfunction calculated using the eGFR_CKD-EPI_ and eGFR_CG-BSA_ equations (Fig. 2).

Based on these results, we used the eGFR_MDRD4_ formula to analyze the incremental prognostic value of renal dysfunction in acute PE patients.

### Risk stratification improvement with adding eGFR_MDRD4_

**Fig. 3:**
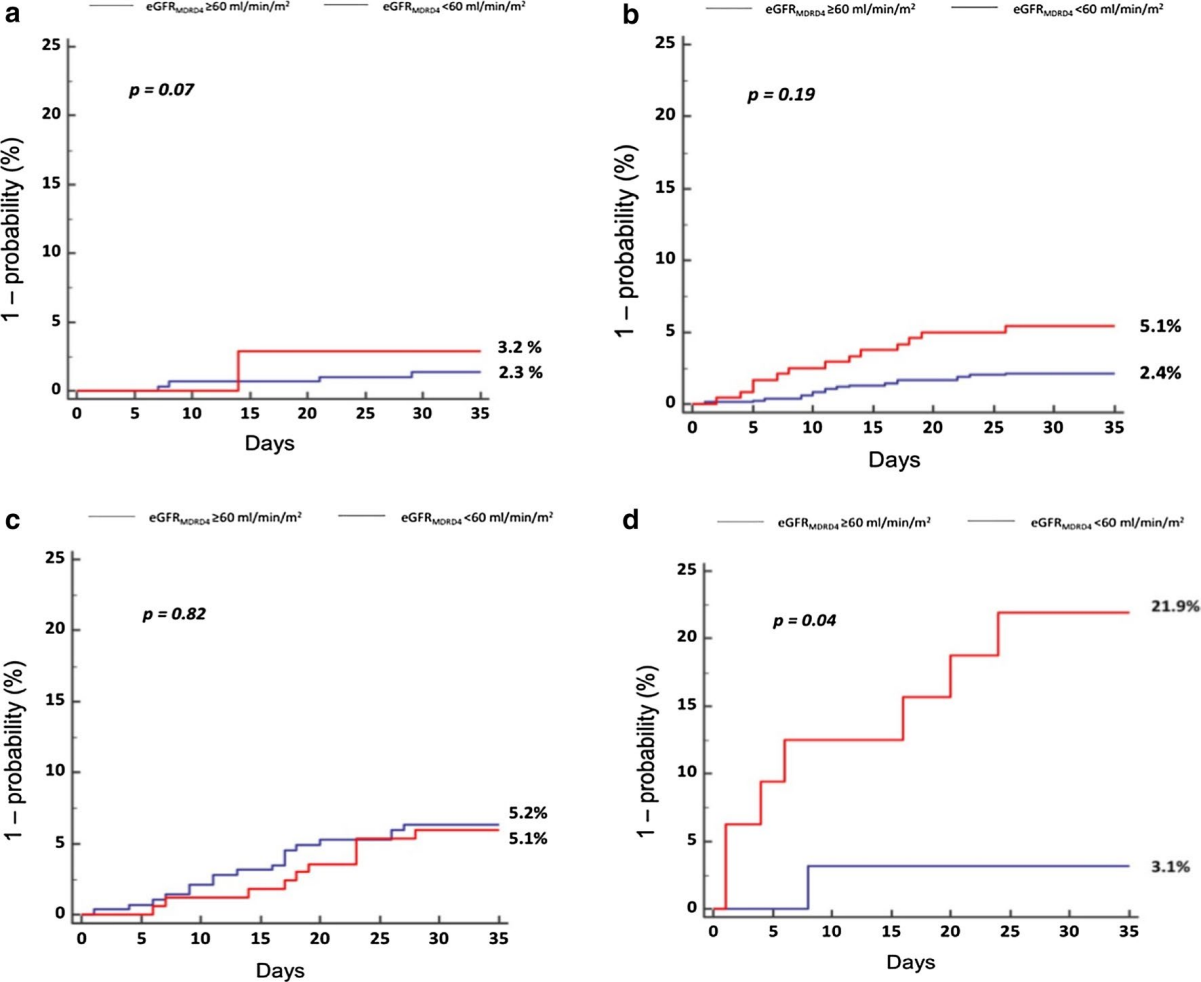
Competing risk cumulative incidence of International Society of Thrombosis and Haemostasis-defined bleeding events at 30 days in low-risk (**a**), intermediate–low-risk (**b**), intermediate–high-risk (**c**), and high-risk patients (**d**) with acute pulmonary embolism stratified by renal function estimated with the four-variable Modification of Diet in Renal Disease equation. eGRF_MDRD4_: estimated glomerular filtration rate calculating using the four-variable Modification of Diet in Renal Disease formula

In total, 28 low-risk patients (1.4%) were managed with outpatient treatment (discharged from emergency room to home) and 198 patients (10.2%) had early discharge on day one after PE diagnosis, without any significant difference between eGFR_MDRD4_-defined function groups (Table 1). High-risk PE was mainly treated with systemic thrombolysis. The rate of use of advanced therapies (i.e., systemic thrombolysis, surgical embolectomy, or extra-corporeal membrane oxygenation) did not differ between patients with and those without eGFR_MDRD4_-defined renal dysfunction across each ESC risk category (Additional file 1: Table S4). The higher rate of bleeding observed in patients with eGFR_MDRD4_ < 60 ml/min was driven by a significantly higher crude incidence of bleeding in high-risk patients with renal dysfunction (Fig. 3).

**Fig. 4:**
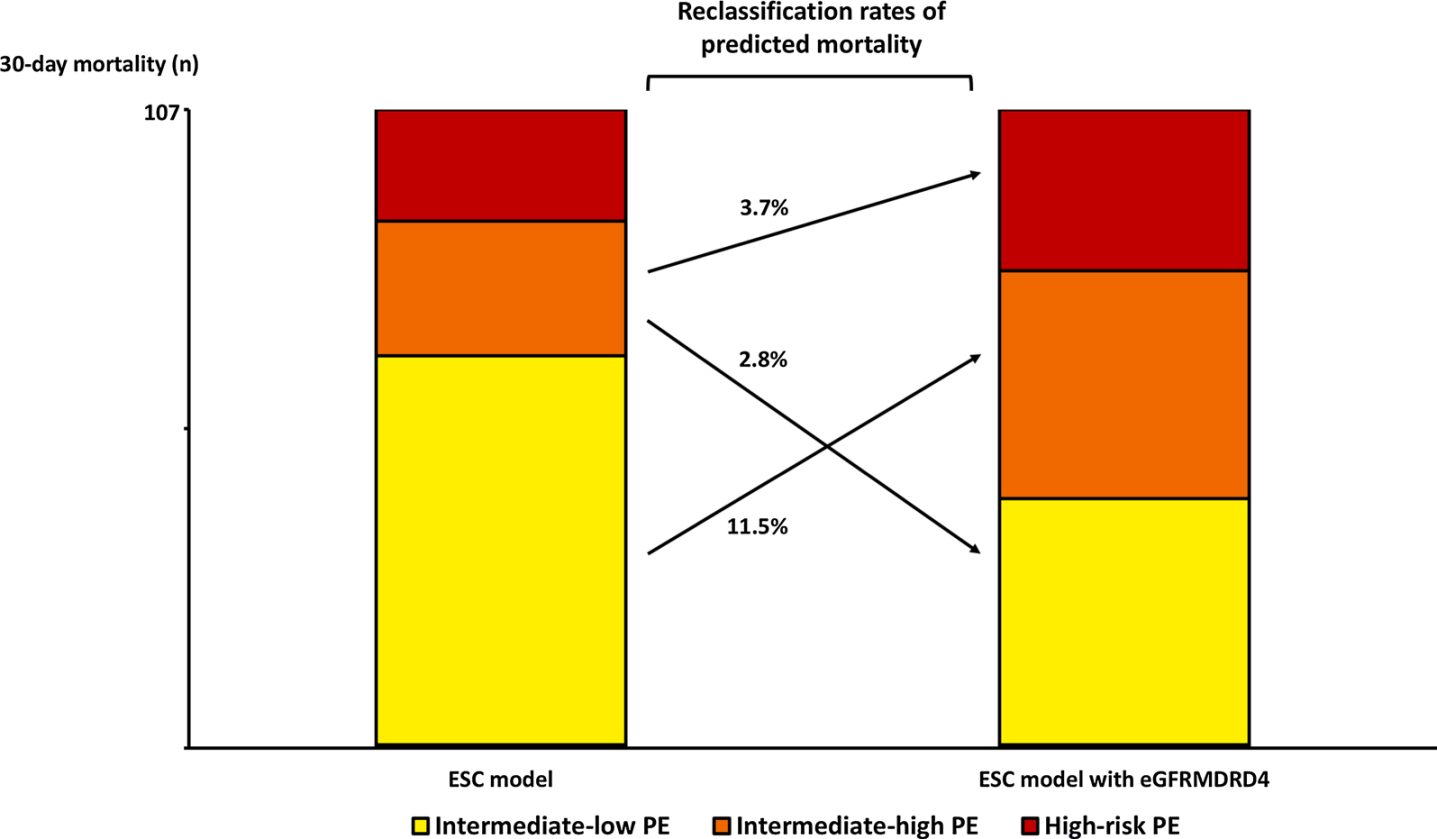
Reclassification of predicted mortality across risk categories between the ESC model and the ESC model with eGFR_MDRD4_-defined renal dysfunction in the study population. ESC: European Society of Cardiology

Patients with renal dysfunction had a higher observed mortality rate than patients with normal kidney function. This difference was not constant across each prognostic category (p for interaction = 0.05). The observed mortality rate was significantly higher for intermediate–low-risk and high-risk PE patients with renal dysfunction as compared to those with normal renal function (Fig. 1). The addition of eGFR_MDRD4_ information on top of the ESC model improved the global model fit with a higher Nagelkerke’s R^2^ and lower Bayes and Akaike information criteria, had better discriminatory capacity with a significant increase in Harrell’s C index (0.631 vs 0.676, *p* = 0.001), and better calibration with an increase in Hosmer–Lemeshow parameters. The IDI, continuous NRI, and user category NRI were significantly increased when adding renal dysfunction to the ESC model, yielding 18% of predicted 30-day death reclassification based on the observed mortality rates with the ESC model as reference (Table 2). Among the 18% of patients reclassified by the addition of eGFR_MDRD4_-defined renal dysfunction, predicted mortality reclassifications across risk categories were as follows: 63.1% from intermediate–low risk to eGFR-defined intermediate–high risk, 15.8% from intermediate–high risk to eGFR-defined intermediate–low risk, and 21.0% from intermediate–high risk to eGFR-defined high risk (Fig. 4).

**Table 2:** Overall model fit, discrimination, calibration indices, and predicted risk reclassification when renal dysfunction (i.e., eGFR_MDRD4_ < 60 ml/min) is added or not to the European Society of Cardiology (ESC) model for the prediction of the 30-day all-cause death after acute pulmonary embolism in the study population and in the RIETE cohort for external validation

	Study population (*n* = 1943)		External validation (*n* = 14,234)	
	ESC model with eGFR_MDRD4_*		ESC model with eGFR_MDRD4_*	
	No	Yes	No	Yes
Models (OR, 95%CI)				
ESC model	2.2 (1.6–2.7)	1.8 (1.4–2.4)	1.9 (1.7–2.1)	1.9 (1.7–2.2)
ESC model with eGFR_MDRD4_*	‒	2.3 (1.5–3.4)	‒	2.1 (1.7–2.6)
Overall model fit				
Bayes information criteria	812.3	804.4	3458.3	3386.5
Akaike information criteria	801.2	787.6	3443.2	3363.2
Nagelkerke’s R^2^	1.6%	2.3%	0.6%	1.2%
Discrimination				
Harrell’s c index	0.631	0.676^#^	0.617	0.667^#^
Calibration				
Adjusted *χ* Hosmer–Lemeshow goodness of fit across deciles of risk	1.15	1.21	18.0	18.2
P Hosmer–Lemeshow	0.009	0.06	< 0.001	0.12
Risk reclassification between ESC model and ESC model with eGFR*				
IDI	1.1%(95% CI 0.5–1.7; *p* < 0.001)		0.5% (95% CI 0.3–0.6; *p* < 0.01)	
Continuous NRI	46.9%(95% CI 27.6–66.2; *p* < 0.001)		45.2% (95% CI 35.1–55.3; *p* < 0.001)	
User category NRI	19.5% (95% CI 6.3–32.6; *p* = 0.004)		–	
% of 30-day mortality correctly reclassified	18%		–	

eGFR, estimated glomerular function; MDRD4, the four-variable Modification of Diet in Renal Disease equation; OR, odds ratio; IDI, integrated discrimination improvement; NRI, net reclassification improvement; CI, confidence interval

*eGFR_MDRD4_ < 60 ml/min

^#^Difference in Harrell’s c indices with *p* value < 0.005

### External validation in RIETE

Among the validation cohort of 14,234 patients from the RIETE registry, mean age was 66.7 ± 16.9 years; 52.9% were women. Mean eGFR_MDRD4_ was 75.6 ± 33.2 ml/min/1.73 m^2^ (Additional file 1: Table S5). A total of 601 patients (4.2%) had high-risk PE, 1831 patients (12.9%) had intermediate–high-risk PE, 8925 (62.7%) had intermediate–low-risk PE, and 2877 (20.2%) had low-risk PE (Fig. 1). Patients included in the RIETE registry differed from those included in the study population in terms of preexisting medical conditions, and other relevant clinical, echographic, and laboratory parameters (Additional file 1: Table S5).

The rate of 30-day all-cause death in the RIETE registry was 2.7% (95% CI 2.4–3.0) (384/14,234 patients). The unadjusted observed mortality rate was significantly higher in patients with eGFR_MDRD4_-defined renal dysfunction across each risk category stratified (Fig. 1). Overall global fit and calibration capacities were better when renal dysfunction was added to ESC model in the RIETE population. The Harrel’s C index increased from 0.617 with the ESC model alone to 0.667 with the ESC model including the eGFR_MDRD4_-defined renal dysfunction (*p* = 0.001). Reclassification parameters including the IDI and the continuous NRI were significantly improved in the ESC model with eGFR_MDRD4_ (*p* < 0.01 and *p* < 0.001, respectively) (Table 2).

## Discussion

In our cohort analyses, we accurately demonstrated the incremental value of adding eGFR_MDRD4_-defined renal dysfunction (i.e.,  < 60 ml/min according to KDIGO guidelines [13]) on the top of the prognostic algorithm with improvement of global fit, calibration, and discrimination yielding significant predicted risk reclassification within the intermediate- and high-risk PE categories. Results were confirmed in over 14,000 patients from the multinational RIETE registry, underlining the validity of our findings. Moreover, we confirmed that eGFR_MDRD4_ < 60 ml/min was one of the strongest factors associated with 30-day all-cause death after acute PE with a 2.1-fold increase in the adjusted mortality rate, along with more established predictors including cancer, cardiogenic shock or hypotension, RV dysfunction, and positive troponin.

Our results strengthen those described by Kostrubiec et al. who calculated a continuous NRI of 42% when combining the 2014 ESC risk stratification with renal dysfunction [5]. The additional prognostic value of eGFR_MDRD_ was also observed in 220 patients with non-high-risk PE [22]. In this study, eGFR_MDRD_ < 35 ml/min improved troponin-based risk stratification of acute PE using receiver operating curve-derived parameters. In the present analysis, we used parameters recommended by Cook et al. in robust and well-validated statistical methods to evaluate the additional predictive ability of renal dysfunction [18, 19].

The significantly improved predicted reclassification across risk categories when adding renal function to the prognostic algorithm might warrant potential changes to PE management. First, the addition of eGFR_MDRD4_ < 60 ml/min reclassified some patients from intermediate–low- to intermediate–high-risk PE. Therefore, patients with intermediate–low risk and renal dysfunction could theoretically require careful monitoring in ICU to watch for hemodynamic worsening and administer reperfusion therapy if needed to prevent PE-related death.

Second, we observed reclassification from intermediate–high- to intermediate–low-risk PE when renal dysfunction was taken into account. Patients with intermediate–high-risk PE and normal renal function may benefit from early DOAC prescription, which might enable shorter hospital stay with the use of a single drug approach [23].

Third, some ESC-defined intermediate–high-risk PE patients were stratified as high risk after adding renal dysfunction to the model. Reclassification in these patients could underpin the potential use of a more aggressive reperfusion strategy than anticoagulation alone, but further studies dedicated to patients with renal dysfunction are needed. In a post hoc analysis of a randomized trial evaluating systemic thrombolysis in intermediate–high-risk PE, Barco et al. identified a subgroup of patients who were more likely to have all-cause death, hemodynamic collapse, or recurrent PE (i.e., patients with SBP⩽ 110 mmHg, respiratory rate > 20 breath/min, chronic HF, and active cancer), although some other clinically relevant parameters, notably renal dysfunction, were not documented [24].

The presence of normal renal function should not call into question the need for reperfusion therapy in this PE-risk category. Systemic thrombolysis plus anticoagulation significantly reduced PE-related death as compared to anticoagulation alone (OR, 0.15; 95% CI 0.03–0.78) in a meta-analysis of randomized trials including 2057 high-risk PE [25]. However, the safety of systemic thrombolysis in patients with renal dysfunction remains poorly evaluated to date [26], and the high rate of major bleeding in high-risk patients who received thrombolysis observed in our analysis calls for caution. This might be especially true in elderly patients. Age over 75 years was found to be associated with 30-day bleeding in our cohort study. Data from the RIETE registry including 1172 patients with confirmed PE who received thrombolytic therapy indicated that age > 75 years was a significant predictor of major bleeding through 30 days (OR 2.0; 95% CI 1.2–3.4) [27]. Moreover, patients aged more than 65 years or with kidney disease had a higher risk of intracranial hemorrhage as compared to younger patients or with normal renal function (*p* < 0.001) among 49,500 patients treated with thrombolytic therapy for PE [28]. The bleeding risk of patients with renal dysfunction who were treated with systemic thrombolysis could potentially be overcome by the use of alternative reperfusion strategies, such as ultrasound-facilitated catheter-based therapy (CDT), or surgical embolectomy, which have been associated with lower bleeding rates in unselected populations (absolute risk of bleeding 9.9% with systemic thrombolysis; 5.5% with CDT; 5.0% with surgical embolectomy) [25, 29, 30].

Conversely, adding eGFR_MDRD4_ on top of the ESC prognostic algorithm did not result in any reclassification among the low-risk category.

The strengths of the present study include the prospective patient recording in different centers, the high rate of consecutive inclusions and data completeness (92.4%), the adjudication of clinical end-points, the robustness of statistical approaches as well as the external validation in a large database. In contrast, we were not able to directly test the impact on PE management of the significant predicted risk reclassification when adding renal dysfunction to the ESC algorithm. Nevertheless, we believe that our results shed new light on renal dysfunction in PE that could help to generate clinically relevant hypotheses and provide the background for the design of future studies, especially interventional clinical trials, in which the ESC algorithm might be powerless to define intermediate- or high-risk PE. In this context, Tapson et al. recently specified further parameters besides the ESC-defined risk model, to better stratify intermediate–high-risk PE, including, for example, physical appearance, clot burden on computed tomography or ventilation/perfusion scan, or residual deep vein thrombosis [31]. Finally, the addition of a variable in any risk algorithm, such as the ESC stratification, increases the complexity of assessment and may discourage physicians from using the algorithm in routine practice.

## Conclusion

In conclusion, the addition of eGFR_MDRD4_-derived renal dysfunction on top of the ESC prognostic algorithm led to significant risk reclassifications within the intermediate- and high-risk PE categories. The impact of risk stratification integrating renal dysfunction on therapeutic management for acute PE requires further studies.

## Supplementary Information

**Additional file 1**. Table 1 presents the equations for CKD-EPI, MDRD4, and BSA-CG for the calculation of estimated glomerular filtration rate. Table 2 presents the baseline characteristics and management of the study population according to eGFR calculated with the eGFRCKD-EPI and eGFRCG-BSA formulae. Table 3 presents unadjusted risk predictors of 30-day all-cause mortality and 30-day bleeding. Table 4 presents in-hospital therapies with and without renal dysfunction defined by eGFRMDRD4 < 60 ml/min. Table 5 presents the baseline characteristics of the study population (n= 1,943) and the external validation RIETE population (n = 14,234).

## Data Availability

The datasets used and/or analyzed during the current study are available from the corresponding author on reasonable written request.
