# Supplementary material for: Renal dysfunction improves risk stratification and may call for a change in the management of intermediate- and high-risk acute pulmonary embolism: results from a multicenter cohort study with external validation
Source: Crit Care. 2021 Feb 9;25:57. doi: 10.1186/s13054-021-03458-z (PMC7874488; doi:10.1186/s13054-021-03458-z)
Supplement: Supplementary file 1 — Additional file 1. Table 1 presents the equations for CKD-EPI, MDRD4, and BSA-CG for the calculation of estimated glomerular filtration rate. Table 2 presents the baseline characteristics and management of the study population according to eGFR calculated with the eGFRCKD-EPI and eGFRCG-BSA formulae. Table 3 presents unadjusted risk predictors of 30-day all-cause mortality and 30-day bleeding. Table 4 presents in-hospital therapies with and without renal dysfunction defined by eGFRMDRD4 < 60 ml/min. Table 5 presents the baseline characteristics of the study population (n= 1,943) and the external validation RIETE population (n = 14,234). [file 13054_2021_3458_MOESM1_ESM.docx]

***Critical Care***

***Original article***

**Additional File 1**

**Renal dysfunction improves risk stratification and may call for a change in the management of intermediate and high risk acute pulmonary embolism:**

**Results from a multicenter cohort study with external validation**

Romain Chopard, David Jimenez, Guillaume Serzian, Fiona Ecarnot, Nicolas Falvo, Gilles Capellier, François Schiele, Laurent Bertoletti, Manuel Monreal Bosch, Nicolas Meneveau

**Additional Table 1: Equations for CKD-EPI, MDRD4, and BSA-CG for the calculation of estimated glomerular filtration rate**

|  | **Equation** |
| --- | --- |
| **CKD-EPI** | = 141 * min(Scr/κ,1)^α^ * max(Scr/κ, 1)^-1.209^ * 0.993^Age^ * 1.018 [if female] * 1.159 [if black] |
| **MDRD4** | = 175 x Scr -1.154 x age-0.203 x 1.212 (if patient is black) x 0.742 (if female) |
| **CG-BSA** | = {{((l 40–age) x weight)/(72xSCr)}x 0.85 (if female)} x (1.73m^2^/BSA) |

CKD-EPI: The Chronic Kidney Disease Epidemiology Collaboration equation (1); MDRD4: the four variables the Modification of Diet in Renal Disease equation (2); CG-BSA: the body surface area-adjusted Cockcroft-gault equation (3); BSA: body surface area calculated in meters squared using the Mosteller equation (4). Scr is serum creatinine (mg/dL), κ is 0.7 for females and 0.9 for males, α is -0.329 for females and -0.411 for males, min indicates the minimum of Scr/κ or 1, and max indicates the maximum of Scr/κ or 1.

**Additional Table 2: Baseline characteristics, and management, of the study population (n = 1,943) according to the estimated glomerular filtration rate calculated with the Chronic Kidney Disease-Epidemiology (eGFR_CKD-EPI_) formula, and the body surface-adjusted Cockcroft-Gault (eGFR_CG-BSA_) formula.**

| **Variables** | **eGFR_CKD-EPI_ <60 ml/min** | | **p-value** | **eGFR_CG-BSA_ <60 ml/min** | | **p-value** |
| --- | --- | --- | --- | --- | --- | --- |
|  | **Yes**  **(n = 547)** | **No**  **(n = 1,396)** |  | **Yes**  **(n = 736)** | **No**  **(n = 1,207)** |  |
| Age, years | 78.2 ± 11.4 | 63.1 ± 17.1 | <0.001 | 77.3 ± 12.0 | 64.1 ± 17.3 | <0.001 |
| Male (%) | 215 (39.3) | 747 (53.5) | <0.001 | 271 (36.8) | 691 (57.2) | <0.001 |
| BMI (kg/m^2^) | 27.7 ± 5.9 | 27.2 ± 5.9 | 0.07 | 28.0 ± 6.2 | 27.1 ± 5.8 | 0.007 |
| Site of care |  |  |  |  |  |  |
| Conventional ward | 282 (51.5) | 1046 (74.9) | - | 414 (56.2) | 914 (75.6) | - |
| Intensive Care Unit | 265 (48.4) | 350 (25.1) | - | 322 (43.7) | 293 (24.4) | - |
| **Co-morbidities (%)** |  |  |  |  |  |  |
| Coronary disease | 128 (23.4) | 154 (11.0) | <0.001 | 164 (22.3) | 1&8 (9.8) | <0.001 |
| Pulmonary disease/HF | 62 (11.3) | 112 (8.0) | 0.02 | 69 (9.4) | 105 (8.7) | 0.61 |
| Active cancer | 97 (17.7) | 270 (19.3) | 0.41 | 141 (19.0) | 227 (18.8) | 0.90 |
| Prior VTE | 133 (24.3) | 341 (24.4) | 0.95 | 180 (24.5) | 294 (24.4) | 0.96 |
| Prior bleeding | 24 (4.4) | 43 (3.1) | 0.34 | 29 (3.9) | 38 (3.1 | 0.70 |
| Low risk for long-term VTE recurrence (%) | 123 (22) | 363 (26.0) | 0.10 | 164 (22.3) | 322 (26.7) | 0.03 |
| Associated DVT (%) | 228 (42) | 566 (40.5) | 0.64 | 291 (39.5) | 503 (41.7) | 0.35 |
| **Clinical characteristics** |  |  |  |  |  |  |
| HR at admission (bpm) | 89.5 ± 19.2 | 89.7± 19.4 | 0.86 | 89.7 ± 19.5 | 89.6± 19.3 | 0.92 |
| SBP at admission (mmHg) | 136.4 ± 25.9 | 137.9 ± 21 9 | 0.20 | 135.5 ± 26.2 | 138.1 ± 22.0 | 0.04 |
| SaO^2^ (%) | 92.9 ± 5.4 | 94.0 ± 4.9 | <0.001 | 92.9 ± 5.4 | 93.9 ± 5.0 | <0.001 |
| **Biological data** |  |  |  |  |  |  |
| Hemoglobin (g/dL) | 12.9 ± 2.2 | 13.5 ± 3.4 | <0.001 | 12.9 ± 2.2 | 13.5 ± 3.4 | <0.001 |
| Positive troponin | 354 (64.7) | 499 (35.7) | <0.001 | 430 (58.4) | 423 (35.0) | <0.001 |
| **Echo data** |  |  |  |  |  |  |
| sPAP (mmHg) | 47.9 ± 15.3 | 40.3 ± 15.4 | <0.001 | 47.9 ± 15.7 | 40.6 ± 15.3 | <0.001 |
| RV dysfunction* | 281 (51.4) | 519 (37.2) | <0.001 | 338 (45.9) | 542 (37.0) | <0.001 |
| Peak systolic gradient at the tricuspid valve > 30 mmHg | 237 (43.3) | 402 (28.8) | <0.001 | 286 (38.9) | 353 (29.2) | <0.001 |
| End-diastolic RV/LV diameter ≥1.0 in the apical four-chamber view | 124 (22.7) | 167 (12.0) | <0.001 | 124 (16.8) | 167 (13.8) | 0.07 |
| Flattened intraventricular septum | 104 (19.0) | 163 (11.7) | <0.001 | 112 (15.2) | 155 (12.8) | 0.14 |
| TAPSE < 16 mm | 35 (6.4) | 50 (3.6) | 0.006 | 38 (5.2) | 47 (3.9) | 0.18 |
| Right heart thrombus | 10 (1.8) | 16 (1.1) | 0.23 | 13 (1.8) | 13 (1.1) | 0.19 |
| **sPESI (points, Q1-Q3)** | 2 (1-3) | 1 (0-2) | <0.001 | 2 (1-3) | 1 (0-2) | <0.001 |
| **In-hospital treatments (%)** |  |  |  |  |  |  |
| Reperfusion therapy |  |  |  |  |  |  |
| Thrombolysis | 43 (7.9) | 50 (3.6) | <0.001 | 38 (5.2) | 55 (4.6) | 0.54 |
| Surgical embolectomy | 5 (0.9) | 4 (0.3) | 0.06 | 3 (0.4) | 6 (0.5) | 0.77 |
| ECMO | 7 (1.3) | 2 (0.1) | <0.001 | 6 (0.8) | 3 (0.2) | 0.07 |
| Inferior vena cava filter | 3 (0.5) | 5 (0.4) | 0.55 | 3 (0.4) | 5 (0.4) | 0.98 |
| **Outpatient treatment^a^** | 8 (1.4) | 20 (1.4) | 1.0 | 12 (1.6) | 16 (1.3) | 0.69 |
| **Early discharge^b^** | 45 (8.2) | 153 (11.0) | 0.07 | 60 (8.1) | 138 (11.4) | 0.02 |

BMI: body mass index; VTE: venous thromboembolic; DVT: deep vein thrombosis; PE: pulmonary embolism; HR: heart rate; SBP: systolic blood pressure; SaO2: Arterial oxyhemoglobin saturation; RV: right ventricle; LV: left ventricle; sPAP: systolic pulmonary arterial pressure; LV: left ventricle; TAPSE: tricuspid annular plane systolic excursion; ECMO: extracorporeal membrane oxygenation.

^a^ Pulmonary embolism management from the emergency room to home; ^b^ Pulmonary embolism management with discharge at day one.

**Additional Table 3: Unadjusted risk predictors of 30-day all-cause mortality and 30-day bleeding.**

| **Parameters** | **OR** | **95% Confidence interval** | **p-value** |
| --- | --- | --- | --- |
| **30-day mortality** |  |  |  |
| Age > 75 years | 1.29 | 1.13-1.47 | <0.001 |
| BMI (Per quartile) | 1.51 | 1.24-1.83 | <0.001 |
| Prior coronary disease | 2.32 | 1.48-3.63 | <0.001 |
| Active cancer | 5.61 | 3.76-8.36 | <0.001 |
| Concomitant DVT | 1.58 | 1.042-2.41 | 0.03 |
| Hypotension or cardiogenic shock | 5.37 | 2.86-10.07 | <0.001 |
| Positive troponin | 3.04 | 1.99-4.63 | <0.001 |
| Hemoglobin (per quartile) | 1.89 | 1.54-2.31 | <0.001 |
| RV/LV diameter ratio (per decile) | 3.04 | 1.51-6.11 | 0.002 |
| In-hospital bleeding | 5.38 | 2.96-9.85 | <0.001 |
| eGFR_CKD-EPI_ <60 ml/min/m^2^ | 2.77 | 1.87-4.11 | <0.001 |
| eGFR_MDRD4_ <60 ml/min/m^2^ | 2.89 | 1.95-4.29 | <0.001 |
| eGFR_CG-BSA_ <60 ml/min/m^2^ | 2.68 | 1.80-4.0 | <0.001 |
| **30-day bleeding** |  |  |  |
| Age > 75 years | 1.70 | 1.01-2.70 | 0.02 |
| Prior stroke | 2.31 | 1.16-4.62 | 0.01 |
| Hypotension or cardiogenic shock | 3.49 | 1.77-6.85 | <0.001 |
| RV dysfunction | 2.18 | 1.37-3.48 | 0.001 |
| Positive troponin | 2.76 | 1.70-4.49 | <0.001 |
| eGFR_CKD-EPI_ <60 ml/min/m^2^ | 2.77 | 1.73-4.33 | <0.001 |
| eGFR_MDRD4_ <60 ml/min/m^2^ | 2.40 | 1.51-3.82 | <0.001 |
| eGFR_CG-BSA_ <60 ml/min/m^2^ | 2.14 | 1.35-3.38 | 0.001 |

OR: odds ratio; BMI: body mass index; DVT: deep vein thrombosis; RV: right ventricle; LV: left ventricle; eGFR: estimated glomerular function; CKD-EPI: The Chronic Kidney Disease Epidemiology Collaboration equation; MDRD4: the four variables the Modification of Diet in Renal Disease equation; CG-BSA: the body surface area-adjusted Cockcroft-gault equation.

**Additional Table 4: In-hospital therapies stratified according the European Society of Cardiology (ESC) prognostic algorithm in acute pulmonary embolism patients with and without renal dysfunction defined by an estimated glomerular function calculated with the four variables the Modification of Diet in Renal Disease equation (eGFR_MDRD4_) < 60 ml/min.**

| **ESC risk stratification** | **Low risk PE with renal dysfunction** | | **p-value** | **Intermediate-low risk with renal dysfunction** | | **p-value** | **Intermediate-high risk with  renal dysfunction** | | **p-value** | **High risk  with renal dysfunction** | | **p-value** |
| --- | --- | --- | --- | --- | --- | --- | --- | --- | --- | --- | --- | --- |
|  | No (303) | Yes (35) |  | No (844) | Yes (242) |  | No (287) | Yes (168) |  | No (32) | Yes (32) |  |
| Anticoagulant |  |  |  |  |  |  |  |  |  |  |  |  |
| UFH | 17 (5.6) | 7 (20.0) | 0.001 | 386(45.7) | 101 (41.7) | <0.001 | 169 (58.9) | 109 (64.8) | 0.21 | 19 (59.4) | 22 (68.7) | 0.43 |
| LWMH | 127 (41.9) | 9 (25.7) | 0.06 | 178 (21.1) | 97 (40.1) | 0.84 | 108 (37.6) | 85 (50.6) | 0.006 | 11 (34.3) | 9 (28.1) | 0.12 |
| DOAC | 159 (52.5) | 19 (54.3) | 0.38 | 278 (32.9) | 44 (18.1) | 0.001 | 30 (20.7) | 7 (10.3) | 0.06 | 2 (6.2) | 1 (3.1) | 0.62 |
| Thrombolysis | **-** | - | - | - | - | - | 24 (8.4) | 15 (8.9) | 0.87 | 26 (81.2) | 28 (87.5) | 0.72 |
| Surgical embolectomy | - | - | - | - | 0 (0) | - | 1 (3.6) | 3 (1.8) | 0.74 | 1 (3.1) | 2 (6.2) | 0.31 |
| ECMO | - | - | - | - | - | - | 2 (0.7) | 3 (1.8) | 0.28 | 0 (0) | 3 (9.4) | 0.07 |
| IVC filter | 0 (0) | 0 (0) | 1.0 | 5 (0.6) | 1 (0.4) | 0.74 | 0 (0) | 1 (0.6) | 0.19 | 0 (0) | 1 (3.1) | 0.31 |

UFH: Unfractionned heparin; LMWH: low-molecular weight heparin; DOAC: direct oral anticoagulant; ECMO: extracorporeal membrane oxygenation; IVC: Inferior vena cava.

**Additional Table 5: Baseline characteristics of patients included in the study population (n= 1,943) and in the external validation RIETE population (n = 14,234).**

| **Variables** | **Study population**  **(n=1,943)** | **External validation**  **(n = 14,234)** | **p-value** |
| --- | --- | --- | --- |
| Age, years | 67.3 ± 17.1 | 66.7 ± 16.9 | 0.11 |
| Male (%) | 962 (49.5) | 6710 (47.1) | 0.05 |
| BMI (kg/m^2^) | 27.3 ± 5.9 | 28.7 ± 5.9 | <0.001 |
| **Co-morbidities (%)** |  |  |  |
| Pulmonary disease/HF | 174 (9.0) | 3176 (22.3) | <0.001 |
| Active cancer | 367 (18.9) | 1055 (7.4) | <0.001 |
| **Clinical characteristics (%)** |  |  |  |
| HR at admission (bpm) | 89.6 ± 19.3 | 93.3 ± 20.6 | <0.001 |
| SBP at admission (mmHg) | 137.5 ± 23.1 | 128.2 ± 24.6 | <0.001 |
| SaO^2^(%) | 93.6 ± 5.1 | 91.0 ± 6.6 | <0.001 |
| **Biological data (%)** |  |  |  |
| eGFR_MDRD4_ | 80.7 ± 30.7 | 75.6 ± 33.2 | <0.001 |
| Positive biomarkers | 853 (43.9) | 2915 (20.5) | <0.001 |
| **Echo data** |  |  |  |
| sPAP > 30 mmHg | 639 (32.9) | 6592 (46.3) | <0.001 |
| RV dysfunction (%) | 711 (36.6) | 7450 (52.3) | <0.001 |
| **sPESI score** | 1.01 ± 0.9 | 1.6 ± 1.3 | <0.001 |

BMI: body mass index; HF: heart failure; HR: heart rate; SBP: systolic blood pressure; eGFR: estimated glomerular function; MDRD4: the four variables the Modification of Diet in Renal Disease equation; RV: right ventricle; LV: left ventricle; sPAP: systolic pulmonary arterial pressure; sPESI: simplified Pulmonary Embolism Severity Index

**References**

1. Levey AS, Stevens LA, Schmid CH, Zhang YL, Castro AF, 3rd, Feldman HI, et al. A new equation to estimate glomerular filtration rate. Ann Intern Med. 2009;150(9):604-12.

2. Levey AS, Bosch JP, Lewis JB, Greene T, Rogers N, Roth D. A more accurate method to estimate glomerular filtration rate from serum creatinine: a new prediction equation. Modification of Diet in Renal Disease Study Group. Ann Intern Med. 1999;130(6):461-70.

3. Cockcroft DW, Gault MH. Prediction of creatinine clearance from serum creatinine. Nephron. 1976;16(1):31-41.

4. Mosteller RD. Simplified calculation of body-surface area. N Engl J Med. 1987;317(17):1098.
